# Systemic immune‐inflammation index acts as a novel diagnostic biomarker for postmenopausal osteoporosis and could predict the risk of osteoporotic fracture

**DOI:** 10.1002/jcla.23016

**Published:** 2019-08-19

**Authors:** Hong Fang, Hanqing Zhang, Zhi Wang, Zhongming Zhou, Yunjun Li, Lin Lu

**Affiliations:** ^1^ Department of Gynecology Hubei Provincial Hospital of Traditional Chinese Medicine Wuhan Hubei China; ^2^ Department of Osteology Wuhan Hospital of Traditional Chinese Medicine Wuhan Hubei China

**Keywords:** fracture, neutrophil‐to‐lymphocyte ratio, postmenopausal osteoporosis, risk factors, systemic immune‐inflammation index

## Abstract

**Background:**

Postmenopausal osteoporosis (PMOP) is a bone metabolism disorder involving systematic inflammation activation. Blood routine examination is easily available in clinical practice and contains abundant information reflecting the systematic inflammation level. Thus, it is attractive to achieve early diagnosis of PMOP and predict osteoporotic fracture risk just based on the biomarkers in blood routine examination.

**Methods:**

A multi‐centric prospective cohort study was designed and enrolled postmenopausal women from two independent institutions. All participants underwent the dual‐energy X‐ray absorptiometry (DEXA) scanning for diagnosing PMOP. Blood routine examination was conducted, and the key inflammatory biomarkers such as neutrophil‐to‐lymphocyte ratio (NLR) and systemic immune‐inflammation index (SII) were calculated. PMOP patients were followed up to observe osteoporotic fracture and identify the related risk predictors.

**Results:**

A total of 92 participants out of 238 enrolled postmenopausal women were diagnosed with PMOP, with a prevalence of 38.66%. The main risk factors identified for PMOP included older age (OR = 2.06, 95% CI = 1.14‐3.72), longer menopause duration (OR = 3.14, 95% CI = 2.06‐4.79), higher NLR (OR = 2.11, 95% CI = 1.37‐3.25), and higher SII (OR = 3.02, 95% CI = 1.98‐4.61). Besides age and menopause duration, SII ≥834.89 was newly identified as a prominent risk factor for discriminating osteoporotic fracture risk in PMOP patients (HR = 3.66, 95% CI = 1.249‐10.71).

**Conclusion:**

As an easy and economical biomarker calculated from blood routine examination, SII not only acts as a good risk predictor for PMOP diagnosis but also well discriminates the osteoporotic fracture risk, which deserves further investigation and application in clinical practice.

**Abbreviations**
BMD: bone mineral density
BMI: body mass index
CI: confidence interval
DEXA: dual‐energy X‐ray absorptiometry
HR: hazard ratio
LMR: lymphocyte‐to‐monocyte ratio
MCH: mean corpuscular hemoglobin
MCHC: mean corpuscular hemoglobin concentration
NLR: neutrophil‐to‐lymphocyte ratio
OR: odds ratio
PDW: platelet distribution width
PLR: platelet‐to‐lymphocyte ratio
PMOP: postmenopausal osteoporosis
RDW: red blood cell distribution width
SII: systemic immune‐inflammation index

## 1 INTRODUCTION

Postmenopausal osteoporosis (PMOP) is a chronic systematic disorder of bone metabolism, which is characterized by bone loss, microstructure deterioration, and prone to fragility fracture.^1^, ^2^ Osteoporotic fractures, also known as brittle fractures, are different from fractures that result from violent collisions or unexpected blows; it refers to fractures that occur without trauma or minor trauma.^3^ With the aging of the population rapidly increasing, PMOP is becoming prevalent in postmenopausal women in recent years, causing a serious social health problem and heavy economical burdens.^4^, ^5^ To date, early detection of PMOP and intervention with protective measures have been the most effective healthcare strategies in PMOP management. Traditional diagnostic approach for PMOP is largely based on the dual‐energy X‐ray absorptiometry (DEXA) and assessed by bone mineral density (BMD).^2^ However, a great number of postmenopausal women are unaware of PMOP and tend not to receive DEXA scanning until some adverse incidents owing to osteoporosis occur, such as bone pain or even bone fracture. Therefore, it is urgent to identify easy and efficient biomarkers to early recognize PMOP among postmenopausal women.^6^

It has been well established that PMOP pathogenesis is closely related to body immune dysfunction and systematic inflammation activation.^7^, ^8^ Because women would lose the protection of endogenous estrogen after menopause, a mass of inflammatory cytokines increasingly accumulates, such as tumor necrosis factor‐alpha, interleukin (IL)‐6, IL‐12, and IL‐17. These inflammatory cytokines could mediate oxidative stress injury, provoke osteoclast, and enhance bone absorbability, thus gradually leading to skeletal remodeling and PMOP.^9^ Therefore, it is reasonable to resort to systematic inflammatory biomarkers to early recognize PMOP. For instance, some emerging studies suggested blood neutrophil‐to‐lymphocyte ratio (NLR). As a simple peripheral blood index which could reflect the systemic inflammatory level, NLR can well discriminate PMOP among postmenopausal women, even being superior to C reaction protein.^10^, ^11^ However, to date, scarce study further explored if there are more optimal biomarkers other than NLR in blood routine examination for diagnosing PMOP, and if there are any blood biomarkers could predict fracture risk among PMOP patients.

Given that blood routine examination is easily available, economical and contains abundant useful parameters, it should not be underutilized in PMOP diagnosis and management, which deserves to be further explored. Thus in this study, we established a multi‐centric cohort consisting of postmenopausal women provided with high‐quality data. We mainly aimed to (a) identify more optimal and novel blood biomarkers besides NLR for diagnosing PMOP among postmenopausal women and (b) first explore biomarkers based on blood routine examination for predicting osteoporotic fracture among Chinese PMOP patients.

## 2 MATERIALS AND METHODS

### 2.1 Study participants

This study was conducted with a prospective cohort design and enrolled participants from two independent medical institutions (Hubei Provincial Hospital of Traditional Chinese Medicine and Wuhan Hospital of Traditional Chinese Medicine). The enrolled study participants were postmenopausal women older than 45 years old who had natural menopause for at least 1 year. Participants who had characteristics as follows were excluded: (a) participants who had endocrine or metabolic diseases, such as diabetes mellitus, thyroid or parathyroid diseases, and rheumatism; (b) participants who received calcium supplements or glucocorticoids; (c) participants who had clinical manifestations indicating recently acute or chronic infections; (d) participants who had solid or hematological malignancies; (e) participants who had obvious hepatorenal dysfunctions; and (f) participants who had incomplete information regarding clinical examinations. Ultimately, a total of 238 postmenopausal women were consecutively enrolled between January 2015 and January 2017. There were 154 participants from Wuhan Hospital of Traditional Chinese Medicine and 84 participants from Hubei Provincial Hospital of Traditional Chinese Medicine, respectively. There were no significant differences regarding age, menopause duration, body mass index (BMI), and BMD between the participants from the two institutions. This study was approved by the Ethics Committees of the institutions. All participants were fully informed of this study and given the written consent for participation.

### 2.2 Clinical examinations and follow‐up

All participants enrolled in this study underwent the DEXA scanning (HOLOGIC DISCOVERY A). The BMD values of the lumbar spine 2‐4 and neck of femur were evaluated. BMD values were presented as mineral amount (g) per scanned area (cm^2^) and then transformed into *T*‐scores based on corresponding coefficients. According to the PMOP diagnosis criteria defined by the World Health Organization,^12^ the participants with a *T*‐score ≤−2.5 were divided into the PMOP patients, while the participants with a *T*‐score ≥−1 were divided into the normal group, and the others with −2.5 ≤*T*‐score ≤−1 were divided into the osteopenia group.

In order to obtain a comprehensive blood routine examination, venous blood samples about 6 mL were collected from all participants after overnight fasting. Then, the blood samples were soon sent to the department of clinical laboratory in our hospitals and tested by automatic blood cell analyzer. Blood parameters, such as albumin, neutrophil counts, lymphocyte counts, monocyte counts, platelet counts, platelet distribution width (PDW), mean corpuscular hemoglobin (MCH), mean corpuscular hemoglobin concentration (MCHC), and red blood cell distribution width (RDW), were all recorded. NLR, platelet‐to‐lymphocyte ratio (PLR), and lymphocyte‐to‐monocyte ratio (LMR) were calculated. Systemic immune‐inflammation index (SII) was defined as platelet counts × neutrophil counts/lymphocyte counts.^13^ All parameters were then transformed into categorical variables based on the mean or median value.

All participants’ baseline and demographic data such as age, menopause duration, and BMI were collected at the time of the enrollment. PMOP patients were subsequently followed up every 4 months by telephone or outpatient visit. Osteoporotic fracture was defined as the fracture caused by the decrease in bone density and bone quality after suffering from osteoporosis, which is a pathological fracture and the most serious consequence of osteoporosis. The status of osteoporotic fracture and the corresponding time was inquired and recorded. The end of the follow‐up was January 2019, and all the enrolled PMOP patients were followed up at least for 2 years.

### 2.3 Statistical analysis

All statistical analyses and graphics were conducted in SPSS 22.0 and GraphPad Prism 7.0. Continuous data were expressed as mean ± standard deviation (SD) and examined by Student's *t* test. Categorical data were expressed as absolute number with percentage and examined by the chi‐square test. Univariate logistic analysis was employed to preliminarily screen the potential risk factors for PMOP. Factors with a *P* value less than .05 in the univariate analysis were sent into a forward stepwise multivariate logistic analysis to identify independent risk factors for PMOP. Odds ratio (OR) or hazard ratio (HR) with 95% confidence interval (CI) was used for measuring the strength of the association. Kaplan–Meier curve was depicted to explore the association of blood markers with bone fracture. The Cox proportional hazard regression analysis was used to identify the independent risk factors for fracture. All *P* values were two‐sided, and *P* < .05 was considered statistically significant.

## 3 RESULTS

### 3.1 Participants’ baseline characteristics

The baseline characteristics of the study participants were presented in Table 1. Among the 238 postmenopausal women, 92 patients were diagnosed as PMOP, with the PMOP prevalence of 38.66%. The average age of the PMOP patients (67.2 ± 7.2 years) was significantly older than that of the osteopenia participants (57.3 ± 8.6 years) and the normal participants (54.9 ± 7.9 years) (*P* < .05). The menopause duration significantly increased from the normal group (6.7 ± 4.3 years) to the osteopenia group (11.8 ± 5.1 years) and then to the PMOP group (18.4 ± 6.9 years) (*P* < .05). The BMI of the normal group (24.9 ± 1.1 kg/m^2^) was significantly higher than that of the PMOP group (22.0 ± 1.3 kg/m^2^) (*P* < .05). Both BMD and *T*‐score presented expected significant differences among the three groups (*P* < .05).

**Table 1:** Baseline characteristics of the study participants

Parameters	All participants (n = 238)	Normal group (n = 72)	Osteopenia group (n = 74)	PMOP group (n = 92)	*P* value‐1	*P* value‐2
Age (year)	60.5 ± 8.1	54.9 ± 7.9	57.3 ± 8.6	67.2 ± 7.2	.183	.002*
Menopause duration (year)	12.3 ± 3.1	6.7 ± 4.3	11.8 ± 5.1	18.4 ± 6.9	.023*	<.001*
BMI (kg/m^2^)	23.4 ± 1.1	24.9 ± 1.1	24.1 ± 1.3	22.0 ± 1.3	.894	.010*
BMD (g/cm^2^)	0.71 ± 0.12	0.89 ± 0.17	0.80 ± 0.15	0.59 ± 0.11	.021*	<.001*
*T*‐score	−2.68 ± 0.16	−0.87 ± 0.15	−2.12 ± 0.21	−4.13 ± 0.19	<.001*	<.001*
Albumin (g/L)	42.8 ± 3.8	44.3 ± 3.3	44.7 ± 4.2	40.1 ± 5.4	.385	<.001*
Neutrophil (10^9^/L)	4.80 ± 2.59	4.45 ± 2.47	4.71 ± 2.44	5.23 ± 2.64	.062	.004*
Lymphocyte (10^9^/L)	1.63 ± 0.55	1.66 ± 0.51	1.62 ± 0.61	1.59 ± 0.59	.386	.271
Monocyte (10^9^/L)	0.44 ± 0.13	0.42 ± 0.11	0.43 ± 0.15	0.46 ± 0.14	.823	.505
Platelet (10^9^/L)	227.56 ± 88.91	225.41 ± 87.16	226.63 ± 86.69	232.92 ± 88.98	.069	.038*
PDW (%)	10.98 ± 1.41	11.75 ± 1.61	11.08 ± 1.28	8.73 ± 1.35	.132	.002*
MCH (pg)	29.37 ± 2.7	30.41 ± 2.4	28.56 ± 3.1	28.69 ± 2.9	.079	.997
MCHC (g/L)	318.25 ± 39.03	321.48 ± 38.41	319.28 ± 40.01	316.28 ± 37.03	.073	.061
RDW (%)	43.93 ± 7.3	44.78 ± 6.5	42.38 ± 7.7	45.71 ± 8.2	.591	.048*

Note

*P* value‐1: osteopenia group vs normal group; *P* value‐2: PMOP group vs osteopenia group.

Abbreviations: BMD, bone mineral density; BMI, body mass index; MCH, mean corpuscular hemoglobin; MCHC, mean corpuscular hemoglobin concentration; PDW, platelet distribution width; PMOP, postmenopausal osteoporosis; RDW, red blood cell distribution width.

* Statistically significant.

Postmenopausal osteoporosis patients had a significantly lower albumin concentration than the osteopenia group ([40.1 ± 5.4] vs [44.7 ± 4.2] g/L, *P* < .05). Neutrophil count significantly increased in the PMOP group compared to the osteopenia group ([5.23 ± 2.64] vs [4.71 ± 2.44] ×10^9^/L, *P* < .05). However, there was no statistical significance regarding lymphocyte count and monocyte count among these groups (*P* > .05). There was a slight increase in platelet count between the PMOP group and the osteopenia group ([230.92 ± 88.98] vs [226.63 ± 86.69] ×10^9^/L, *P* < .05). The mean PDW value of the PMOP group was significantly lower than that of the osteopenia group ([8.73 ± 1.35] vs [11.08 ± 1.28] %, *P* < .05), while the mean RDW value of the PMOP group was significantly higher than that of the osteopenia group ([45.71 ± 8.2] vs [42.38 ± 7.7] %, *P* < .05). Neither MCH nor MCHC had statistical significance among the PMOP group, the osteopenia group, and the normal group (*P* > .05).

### 3.2 Risk factors for PMOP patients

Univariate analyses were conducted to identify the parameters associated with PMOP diagnosis, and the results were presented in Table 2. There were more women older than 60 years old (66.3%) in the PMOP patients than that in the non‐PMOP participants (33.6%), and the difference had statistical significance (*P* < .05). PMOP patients were more likely to have menopause over than 12 years compared to non‐PMOP participants (62.0% vs 26.0%, *P* < .05). PMOP patients tend to have lower BMI values (<23 kg/m^2^) compared to non‐PMOP participants (58.7% vs 39.7%, *P* < .05). Similarly, there were more women with lower albumin level (<42 g/L) in the PMOP group (68.5%) than in the non‐PMOP group (45.9%). Higher NLR was more frequently found in the PMOP group (75.0%) than in the non‐PMOP group (41.8%), so was the higher PLR. On the contrary, higher LMR was less frequently found in the PMOP group (35.9%) than in the non‐PMOP group (56.2%). Higher SII was more frequently found in the PMOP group (73.9%) than in the non‐PMOP group (35.6%). Higher PDW was less frequently found in the PMOP group (41.3%) than in the non‐PMOP group (56.8%). However, MCH and MCHC failed to discriminate PMOP among the postmenopausal women, with no significant difference between the PMOP group and the non‐PMOP group (both *P* > .05). Higher RDW was more frequently found in the PMOP group (62.0%) than in the non‐PMOP group (48.6%).

**Table 2:** Univariate logistical regression analysis of risk factors for PMOP among postmenopausal women

Parameters	Non‐PMOP group (n = 146)	PMOP group (n = 92)	Crude OR (95% CI)	*P* value
Age (year)				
<60	97 (66.4%)	31 (33.7%)	1.0	
≥60	49 (33.6%)	61 (66.3%)	3.90 (2.24‐6.77)	<.001*
Menopause duration				
<12	108 (74.0%)	35 (38.0%)	1.0	
≥12	38 (26.0%)	57 (62.0%)	4.63 (2.64‐8.11)	<.001*
BMI (kg/m^2^)				
<23	58 (39.7%)	54 (58.7%)	1.0	
≥23	88 (60.3%)	38 (41.3%)	0.46 (0.27‐0.79)	.004*
Albumin (g/L)				
<42	67 (45.9%)	63 (68.5%)	1.0	
≥42	79 (54.1%)	29 (31.5%)	0.39 (0.23‐0.68)	.001*
NLR				
<3.64	85 (58.2%)	23 (25.0%)	1.0	
≥3.64	61 (41.8%)	69 (75.0%)	4.18 (2.35‐7.43)	<.001*
PLR				
<161.94	75 (51.4%)	34 (37.0%)	1.0	
≥161.94	71 (48.6%)	58 (63.0%)	1.80 (1.06‐3.07)	.030*
LMR				
<4.16	64 (43.8%)	59 (64.1%)	1.0	
≥4.16	82 (56.2%)	33 (35.9%)	0.44 (0.26‐0.75)	.002*
SII				
<834.89	94 (64.4%)	24 (26.1%)	1.0	
≥834.89	52 (35.6%)	68 (73.9%)	5.12 (2.88‐9.11)	<.001*
PDW (%)				
<10.98	63 (43.2%)	54 (58.7%)	1.0	
≥10.98	83 (56.8%)	38 (41.3%)	0.53 (0.32‐0.91)	.019*
MCH (pg)				
<29.37	72 (49.3%)	51 (55.4%)	1.0	
≥29.37	74 (50.7%)	41 (44.6%)	0.78 (0.46‐1.32)	.358
MCHC (g/L)				
<318.25	69 (47.3%)	48 (52.2%)	1.0	
≥318.25	77 (52.7%)	44 (47.8%)	0.82 (0.49‐1.39)	.460
RDW (%)				
<43.93	75 (51.4%)	35 (38.0%)	1.0	
≥43.93	71 (48.6%)	57 (62.0%)	1.72 (1.01‐2.93)	.045*

Abbreviations: BMI, body mass index; CI, confidence interval; LMR, lymphocyte‐to‐monocyte ratio; MCH, mean corpuscular hemoglobin; MCHC, mean corpuscular hemoglobin concentration; NLR, neutrophil‐to‐lymphocyte ratio; OR, odds ratio; PDW, platelet distribution width; PLR, platelet‐to‐lymphocyte ratio; PMOP, postmenopausal osteoporosis; RDW, red blood cell distribution width; SII, systemic immune‐inflammation index.

* Statistically significant.

As presented in Table 3, in the subsequent multivariate analysis, age older than 60 years was identified as an independent risk factor for PMOP diagnosis (adjusted OR = 2.06, 95% CI = 1.14‐3.72). Menopause duration over than 12 years conferred postmenopausal women a high risk for PMOP (adjusted OR = 3.14, 95% CI = 2.06‐4.79), while BMI conferred postmenopausal women a low risk for PMOP (adjusted OR = 0.75, 95% CI = 0.61‐0.92). NLR was confirmed to be an independent risk factor for PMOP diagnosis (adjusted OR = 2.11, 95% CI = 1.37‐3.25). SII was newly identified as an independent risk factor which closely determined PMOP (adjusted OR = 3.02, 95% CI = 1.98‐4.61). RDW also exerted a risk effect mildly indicating PMOP (adjusted OR = 1.29, 95% CI = 1.04‐1.60).

**Table 3:** Multivariate logistical regression analysis of risk factors for PMOP among postmenopausal women

Parameters	Regression coefficient	Adjusted OR (95% CI)	*P* value
Age (year)			
<60		1.0	
≥60	0.78	2.06 (1.14‐3.72)	<.001*
Menopause duration			
<12		1.0	
≥12	1.32	3.14 (2.06‐4.79)	<.001*
BMI (kg/m^2^)			
<23		1.0	
≥23	‐0.07	0.75 (0.61‐0.92)	<.001*
NLR			
<3.64		1.0	
≥3.64	0.84	2.11 (1.37‐3.25)	<.001*
SII			
<834.89		1.0	
≥834.89	1.11	3.02 (1.98‐4.61)	<.001*
RDW (%)			
<43.93		1.0	
≥43.93	0.06	1.29 (1.04‐1.60)	.018*

Abbreviations: BMI, body mass index; CI, confidence interval; NLR, neutrophil‐to‐lymphocyte ratio; OR, odds ratio; RDW, red blood cell distribution width; SII, systemic immune‐inflammation index.

* Statistically significant.

### 3.3 Risk factors for osteoporotic fracture in PMOP patients

**Figure 1:**
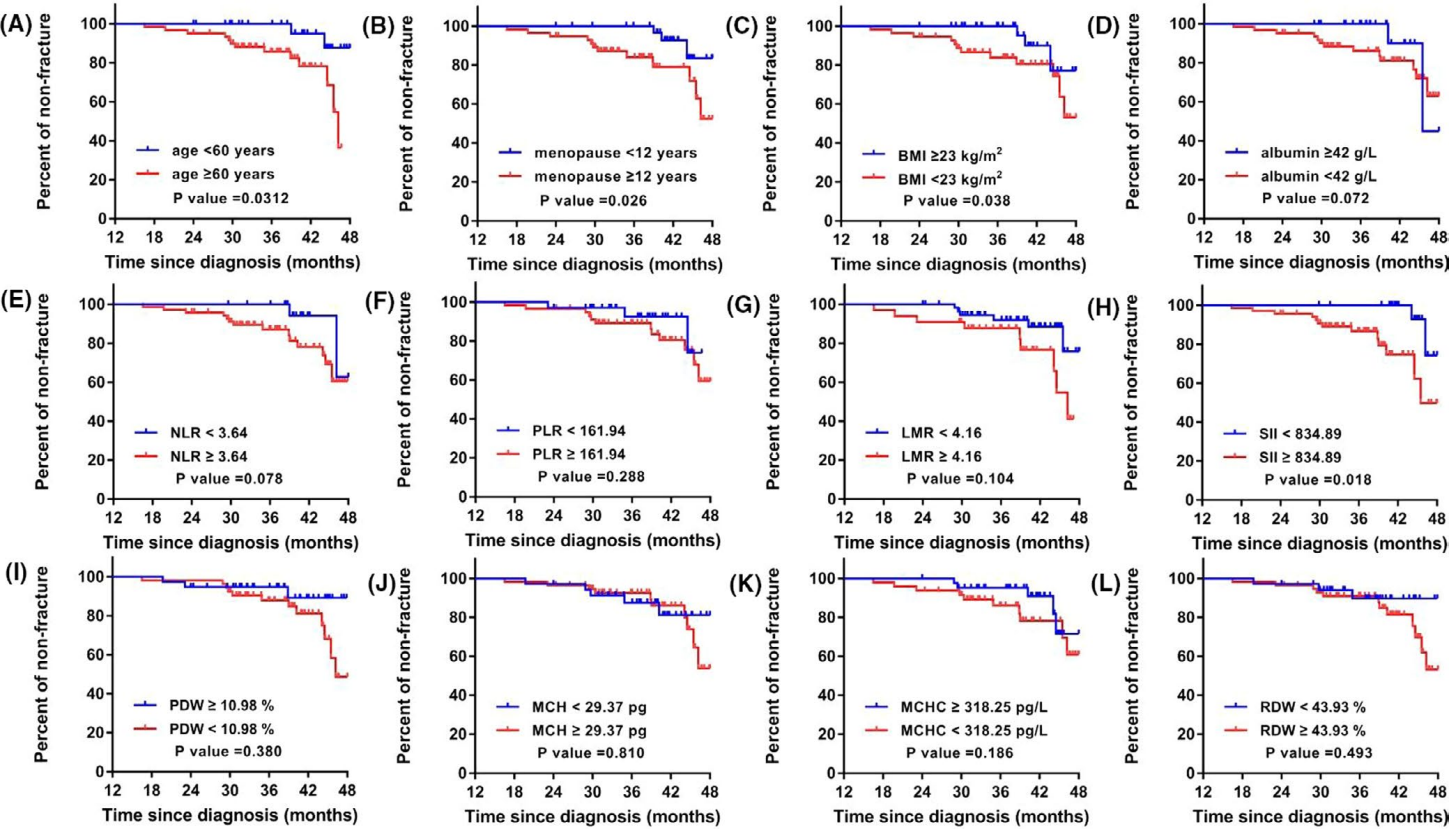
The Kaplan‐Meier curves showing the different biomarkers for discriminating osteoporotic fracture risk in PMOP patients. (A) age for osteoporotic fracture risk; (B) menopause duration for osteoporotic fracture risk; (C) BMI for osteoporotic fracture risk; (D) albumin level for osteoporotic fracture risk; (E) NLR level for osteoporotic fracture risk; (F) PLR level for osteoporotic fracture risk; (G) LMR level for osteoporotic fracture risk; (H) SII level for osteoporotic fracture risk; (I) PDW level for osteoporotic fracture risk; (J) MCH level for osteoporotic fracture risk; (K) MCHC level for osteoporotic fracture risk; (L) RDW level for osteoporotic fracture risk

The median follow‐up time was 38.8 (31.7‐43.4) months. During this period, 15 PMOP patients happened to osteoporotic fracture, with an incidence rate of 16.30% (15/92). In the 77 patients with censored survival data, 12 patients were censored because of loss of contact or withdraw, 65 patients were censored because the outcome event did not occur until the end of the follow‐up. As displayed in Figure 1, PMOP patients with age ≥60 years significantly tend to have osteoporotic fracture than those with age <60 years (*P* < .05; Figure 1A). PMOP patients with duration of menopause ≥12 years were also more likely to occur fracture (*P* < .05) (Figure 1B). High BMI ≥ 23 kg/m^2^ can play a protective effect on PMOP patients against fracture risk (Figure 1C). Although lower albumin level and higher NLR level seemed to increase the risk of fracture, the differences were not adequately achieve statistical significance (both *P* > .05; Figure 1D,E). SII displayed an excellent ability to discriminate high fracture risk patients or low fracture risk patients (*P* < .05; Figure 1H). The other blood routine biomarkers, such as PDW, MCH, MCHC, and RDW, were shown to have no significant differences regarding fracture risks (all *P* > .05; Figure 1I‐L, respectively).

In the subsequent hazard analysis, the HR of age ≥60 years for osteoporotic fracture was 3.65 (95% CI = 1.282‐10.41). The HR of menopause duration ≥12 years for osteoporotic fracture was 2.88 (95% CI = 1.014‐8.188). The HR of SII ≥ 834.89 for osteoporotic fracture was 3.66 (95% CI = 1.249‐10.71), which exerted a significant risk predictive role for osteoporotic fracture in PMOP patients.

## 4 DISCUSSION

Body normal bone formation and function are known for depending on a dynamic balance process, in which osteoblasts induce osteogenesis while osteoclasts induce bone resorption. When women reach menopause, a series of complicated biological changes happens due to aging, calcium lost, and estrogen falling, including inflammatory microenvironment activation and immune system hypofunction.^14^ These changes would significantly impact the women bone microstructure no matter in local or systemic way, because multiple immune cells, especially B lymphocytes which are responsible for mediating the humoral immune response, reside in the bone marrow cavity. The dysfunctional lymphocytes could initiate the cascade of inflammatory cytokines and chemokines, and provoke neutrophil and macrophage aggregation.^15^, ^16^ The dynamic balance of bone formation was thus broken, generally inclining to the side of osteoclast‐induced bone resorption. This inflammatory and immune imbalance could cause bone mass loss and weaken the bone intensity, correspondingly shaping PMOP and predisposing women to fracture.^17^

Over the past decade, increasing studies have reported that many blood routine examination‐derived biomarkers, such as NLR, PLR, and LMR, whose levels could be closely related to systemic inflammation and immune response status.^18^ These biomarkers are proved to be well associated with various infectious diseases, oncological diseases and autoimmune diseases.^19^, ^20^, ^21^ In recent years, following NLR, PLR, and LMR, SII has been discovered as an emerging indicator for these diseases and shed great predictive and diagnostic potential.^22^ However, to date, whether SII could also help to determine PMOP risk in postmenopausal women remains largely unclear. In the present study, our results first revealed higher SII could confer postmenopausal women an obviously higher risk of suffering PMOP. According to the adjusted ORs evaluated in our analysis, the risk of PMOP caused by the higher SII is nearly equivalent to the risk caused by the long menopause duration, indicating that the up‐regulated SII can be regarded as a strong indicator for PMOP diagnosis. Considering that DEXA scanning is a relatively expensive and radioactive examination, sometimes postmenopausal women might have poor adherence to receive it.^23^ Compared to DEXA scanning, SII could be easily and economically obtained from blood routine examination. Therefore, in the future clinical practice, clinicians might resort to SII to screen PMOP high‐risk population in postmenopausal women in combination of her age and menopause duration. SII might be a very useful biomarker to help determine PMOP risk.

Osteoporotic fracture is an unfavorable complication of PMOP, which would seriously impair PMOP patients’ quality of life and survival.^24^ Early and accurate fracture risk assessment remains an important topic in the management of PMOP.^25^, ^26^ Although the World Health Organization developed a tool of Fracture Risk Assessment (FRAX) to generally predict the fracture probability during 10 years,^27^ it is still necessary to establish an individualized risk estimation aiming at the specific PMOP patients in a specific institution. In this study, we investigated multiple blood routine biomarkers in relation to the risk of fracture. We found although many inflammatory biomarkers, such as NLR and RDW, could well recognize the risk of PMOP in postmenopausal women, they failed to further discriminate the risk of osteoporotic fracture in PMOP patients. SII is an emerging inflammatory index which can overall reflect the body immune and inflammatory status. Given that platelet, neutrophil, and lymphocyte might be easily influenced by individual differences, such as age, gender, and comorbidity, SII is constructed by the ratio of these indices and could attenuate the individual interferences.y^18^, ^28^ Noteworthily, only SII remained to exert an effective role in discriminating high or low fracture risk in the follow‐up. This good performance of SII might be because it integrated three immune or inflammatory indices including platelet counts, neutrophil counts, and lymphocyte counts. Thus, it can comprehensively and stably reflect a landscape of PMOP.

Although the novel findings as mentioned above, there were inevitably some limitations in our study. First, although we enrolled a great number of postmenopausal women as the participants, the number of our target population, namely PMOP patients, was still not abundant. The small sample size of our target population may restrict some further deep and meaningful subgroup analysis. Second, the follow‐up time in our study seemed to be not adequately long to observe the outcome event of osteoporotic fracture incidence. Only a few PMOP patients occurred fracture until the endpoint, and this might lead to some fluctuation of the estimated HR values. Therefore, in the future, more PMOP patients should be enrolled and long followed in order to yield more meaningful and insightful findings. Third, PMOP is a systemic disease involving in multiple body disorders. Currently, we have adequately explored the inflammatory signs. However, there must be some other aberrant blood routine signs reflecting the disease. Therefore, in the future, it is attractive and valuable to investigate more markers such as the blood lipid markers and blood coagulation markers in PMOP.

In summary, the present study newly identified that higher SII acts as a significant risk predictor for PMOP diagnosis among postmenopausal women. More than that, SII could also well discriminate the osteoporotic fracture risk in PMOP patients. Because SII is an easy and economical blood routine examination‐derived biomarker, in the future clinical practice, it may play an important role in PMOP screening and prevention.
